# The Effect of HLA Polymorphisms on the Recognition of Gag Epitopes in HIV-1 CRF01_AE Infection

**DOI:** 10.1371/journal.pone.0041696

**Published:** 2012-07-27

**Authors:** Busarawan Sriwanthana, Masahiko Mori, Mari Tanaka, Sei Nishimura, Toshiyuki Miura, Panita Pathipvanich, Pathom Sawanpanyalert, Koya Ariyoshi

**Affiliations:** 1 Department of Medical Sciences, Ministry of Public Health, Nonthaburi, Thailand; 2 Institute of Tropical Medicine, Nagasaki University, Nagasaki city, Nagasaki, Japan; 3 Department of Paediatrics, The Peter Medawar Building for Pathogen Research, University of Oxford, Oxford, United Kingdom; 4 Advanced Clinical Research Centre, Institute of Medical Science, University of Tokyo, Minato-ku, Tokyo, Japan; 5 Day Care Centre, Lampang Hospital, Lampang, Thailand; University of Alabama at Birmingham, United States of America

## Abstract

**Introduction:**

The design of a globally effective vaccine rests on the identification of epitopes capable of eliciting effective cytotoxic T lymphocyte (CTL) responses across multiple HIV clades in different populations. This study aims to discern the effect of HLA polymorphisms and the cross-clade reactivity or clade-specificity of epitopes in Thailand where HIV-1 CRF01_AE is circulating.

**Materials and Methods:**

14 peptides based on consensus HIV-1 CRF01_AE amino acid sequences were designed for use in IFN-γ ELISpot assays and ^51^Cr release assays among 66 HIV-1 CRF01_AE-infected Thai patients. For ELISpot responders carrying HLA alleles currently unknown to restrict CRF01_AE epitopes, *in silico* epitope-HLA prediction was performed.

**Results:**

29/66 (43.9%) patients recognized at least one peptide. In total 79 responses were seen against all 14 peptides. 28/79 (35.4%) of the responses were in patients with HLA alleles previously reported to restrict CRF01_AE epitopes, 24/79 (30.4%) responses were in individuals with HLA alleles previously reported to restrict epitopes of HIV clades other than CRF01_AE, and the remaining 27/79 (34.2%) responses were not associated with HLA alleles previously known to restrict HIV epitopes. *In silico* epitope prediction detected 19 novel, epitope-HLA combinations, and 11/19 (57.9%) were associated with HLA-C alleles. We further confirmed a novel HLA restriction of a previously identified HIV-1 Gag epitope [p24_122–130_: PPIPVGDIY (PY9)] by HLA-B*40:01 with a standard ^51^Cr release assay.

**Discussion:**

CTL recognition sites in HIV-1 Gag were similar among different clades but the HLA restriction differed in Thai patients. This disparity in HLA restriction along different populations illustrated the importance of clade- and population-specific HLA analysis prior to CTL vaccine design.

## Introduction

The control of the Human Immunodeficiency Virus type 1 (HIV-1) epidemic requires the design of a globally effective HIV vaccine. However, the sequence diversity of HIV across clades and the host's human leukocyte antigen (HLA) polymorphism poses a major challenge in the development of a globally effective HIV-1 vaccine capable of inducing cross-clade reactivity [1]–[3]. The design of polyvalent vaccines aimed at inducing HIV-specific cytotoxic T lymphocyte (CTL) responses has been one of the main focuses in the field of HIV vaccinology for several reasons. Firstly, HIV-specific CTLs play a key role in the control of HIV-1 replication during acute infection and in determining the consequent viral set point [4]. Secondly, studies in macaques have shown that vaccine induced recruitment of Simian Immunodeficiency Virus (SIV)-specific CTLs can effectively control viral replication and slow disease progression [5], [6]. Thirdly, unlike neutralizing antibodies, CTLs target proteins such as Gag and Pol, which are relatively conserved across various clades [1], [2], [7]. Currently, there are 13 prototype HIV clades and 43 circulating recombinant forms (CRF) of HIV-1 group M in the world which are of global importance [2]. However, most immunogenicity studies of the CTL epitopes are conducted in the setting of clade B infection in Caucasian cohorts or clade C infection in African or Indian populations (Epitope Maps, Los Alamos database. http://www.hiv.lanl.gov/), and limited information is available on the immunogenicity of CTL epitopes in the CRF01_AE subtype dominating the epidemic in south-east Asian countries such as Thailand. Here there is a unique class I HLA allele distribution and the prevalence of the highly protective HLA allele B*57 is lower than in other ethnicities; 7%–9% among Africans, 5%–7% among Caucasians, and less than 5% among Asians [1].

HLA polymorphisms can also present a challenge in the design of a vaccine. The HLA loci are the most polymorphic genes in the human genome [8]. As of February 2012, 1,757 of class I HLA-A, 2,338 of HLA-B, and 1,304 of HLA-C alleles have been reported in the IMGT/HLA database [9]. The pattern of HLA distribution and their influence on clinical progression differs among ethnic groups [1], [10]–[12]. How this divergence across populations plays an effect on the CTL recognition of HIV-1 peptides is not yet fully elucidated, but understanding this is critical for the development of a universal CTL vaccine which delivers protection across various populations.

In the present study, the extent of T cell cross-reactivity to published HIV-1 CRF01_AE sequences in 66 HIV-1 infected Thai patients was evaluated in *ex vivo* ELISpot assays using 14 peptides encoding the Gag protein of the CRF01_AE sequence. The cross-clade specific T cell responses were further elucidated in a standard chromium release assay. We report here that 43% of CRF01_AE infected individuals reacted to at least one peptide of the CRF01_AE sequences that were tested. In this study we aimed to discern the effect of HLA polymorphisms and the cross-clade reactivity or clade-specificity of epitopes among HIV-1 CRF01_AE infected Thai patients, in order to fill in the missing information on epitopes and HLA alleles in Asia.

## Materials and Methods

### Subjects

This study was approved by Thai Ministry of Public Health Ethics Committee as described elsewhere [13]. Written informed consent was obtained from all patients after explaining the purpose and expected consequences of the study. In case of patients who were school-age, we obtained the written informed consent from their parents as well. 66 HIV-1 CRF01_AE chronically infected patients were recruited at the Lampang hospital, a government referral hospital in northern Thailand. Patients were eligible for inclusion if they were antiretroviral drug naïve at the time of the study. When we attempted to confirm the transmission route, we found one study patient transmitted as an intra-venous drug user (IDU). Thus we excluded this patient from the analysis and corrected the candidate number from 67 to 66. The heterosexual transmission is the predominant mode of HIV transmission in Thailand. It is known that CRF01_AE spread mainly in heterosexually transmitted population. Although rare, subtype B is detected among IDUs [14]–[16]. In our 66 study patients, the transmission route of all infection was confirmed to be heterosexual by direct interview. Furthermore a part of study patients were confirmed to carry a CRF01_AE virus by direct sequencing [13].

### Class I HLA typing

Genomic DNA was extracted from buffy coats using the QIAamp DNA blood Mini Kit (Qiagen, Hilden, Germany) and 4-digit class I HLA typing for A, B and C loci was performed by bead-based array hybridization (WAKFlow HLA typing kit, Wakunaga Pharmaceutical, Hiroshima, Japan) at the Kyoto HLA Laboratory, Kyoto, Japan.

### Synthetic peptides containing previously reported CTL epitopes

A set of 14 HIV-1 CRF01_AE Gag peptides were designed based on CTL epitope regions published in the Los Alamos database at the time this study was planned in the year 2000. Of these, 7 encoded previously reported CRF01_AE epitopes. For the remaining epitopes unreported in CRF01_AE infections, the peptide sequences were altered to fit the dominant CRF01_AE sequence as published in the Los Alamos database. The most predominant sequences among available single isolate sequences were selected to design the peptides. We often extended the peptide length up to 12-mer to maximize the frequency of responses if the extension spanned other epitopes restricted by different allele. p24_131–143_ KRWIILGLNKIVR (KR13) was also included despite 13-mer, as it spans both HLA-B27-restricted KRWIILGLNK (KK10) and HLA-A11 and A3-restricted ILGLNKIVR (IR9). Peptides were synthesized by Sigma Genosys (Hokkaido, Japan) with a high purity of >90% as determined by high-pressure liquid chromatography.

**Figure 1:**
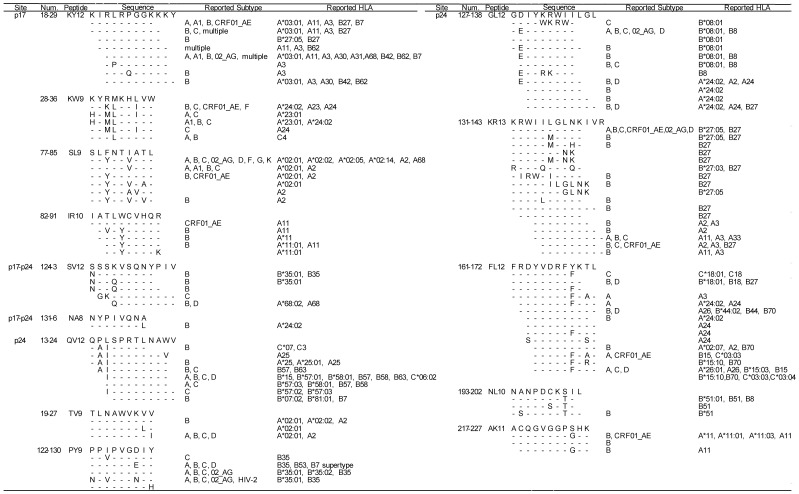
Sequence variants of 14 peptides and their HLA restrictions. Epitope variations of the 14 peptides used in our ELISpot assays reported in the Los Alamos database and their restricting HLA alleles were listed.

We further summarised the optimal epitope sequences of the tested 14 peptides and their variants reported in the Los Alamos database for consequent cross-clade reactivity or clade-specificity analysis (Figure 1). In total, 97 variants of the 14 epitopes have been previously reported from various clades; all of the 14 peptides included reported epitopes from clade B, and 7 of these included epitope reports from CRF01_AE [p17_18–29_: KIRLRPGGKKKY (KY12), p17_28–36_ KYRMKHLVW (KW9), p17_77–85_ SLFNTIATL (SL9), p17_82–91_ IATLWCVHQR (IR10), p24_131–143_ KRWIILGLNKIVR (KR13), p24_161–172_ FRDYVDRFYKTL (FL12) and p24_217–227_ ACQGVGGPSHK (AK11)]. The greatest diversity was found in KR13 with 18 epitope variants, followed by 14 in FL12 and p24_127–138_ GDIYKRWIILGL (GL12). In contrast, p17_131_- p24_6_: NYPIVQNA (NA8) had only one epitope variant, reported from clade B, while p24_19–27_ TLNAWVKVV (TV9) had 3 epitope variants. Almost all of the restricting HLA alleles were derived from HLA-A or HLA-B alleles, and only 6 epitope variants in 3 peptides included HLA-C alleles as their restricting HLA allele. Out of 66 tested patients, 63 (95.5%) patients had at least one of previously reported HLA alleles which were responsible for CRF01_AE epitope recognition: 63 (95.5%) patients with relevant A alleles, 21 (31.8%) with B alleles, and 7 (10.6%) with C alleles.

### Peptide-based IFN-γ ELISpot assay

In ELISpot assay, 14 peptides were tested against all of 66 patients. Peripheral blood mononuclear cells (PBMCs) were isolated by density gradient separation using a Vacutainer CPT Cell Preparation Tube (BD, Franklin Lakes, NJ, USA) and washed twice with RPMI-1640 medium (Sigma-Aldrich, St. Louis, MO, USA). 1×10^5^ fresh PBMCs/well were then plated onto multiScreen plates (MAHA54510; Millipore, Billerica, MA, USA) that had been coated overnight at 4°C with 50 µl of anti-IFN-γ capture Ab 1-D1-K (2 µg/ml; Mabtech, Nacka Strand, Sweden). Peptides were added directly to wells at a final concentration of 1 µM in 50 µl of R10 and incubated at 37°C in 5% CO_2_ for 24 hrs. PBMCs were stimulated with either media alone in negative control wells, 10 µg/ml phytohaemagglutinin (PHA; Sigma-Aldrich) in positive control wells or peptides (1 µM final concentration) for 24 hrs at 37°C. Plates were washed extensively with wash buffer (PBS/Tween20 0.001%), followed by incubation with biotinylated anti-human IFN-γ mAb (0.5 µg/ml; clone 7-B6-1; Mabtech) in PBS/10% FBS for 2 hrs at 37°C. Following six further washes with wash buffer, 2 µg/ml streptavidin HRP (Mabtech) was added to wells and incubated for 1 hr at room temperature. Spots were visualized by adding BCIP/NBT substrate (Millipore) and counted by an independent scientist in a blinded fashion using an automated Enzyme-Linked Immunospot (ELISpot) Plate Reader System with the KS 4.3 software. Positive spot forming units (SFU) were counted and results were expressed as SFU/1×10^6^ PBMCs. A response was considered positive if it was four times higher than the negative control and greater than 55 SFU/1×10^6^ PBMC [17].

### *In silico* epitope prediction

For ELISpot responses induced by patients with HLA alleles previously unknown to restrict the tested CRF01_AE epitopes, we performed *in silico* prediction of HLA restriction elements and epitopes within peptides using the online prediction tool HLArestrictor [18]. For each HLA-epitope combination we defined the binding affinity using the thresholds Strong Binder (SB) with binding affinity ≤50 nM or or % Rank ≤0.5, Weak Binder (WB) with binding affinity ≤500 nM or % Rank ≤2.0, and Combined Binder (CB) with binding affinity ≤500 nM, and % Rank >2.0, according to the original definitions by the program designers [18].

### ^51^Cr release assay

CTL lysis assays were performed using standard ^51^Chromium release assays as previously described [19]. In brief, peptide-specific CTL lines were generated from freshly isolated PBMCs of HIV-1 infected donors. One seventh of the PBMCs were stimulated with PHA (2 µg/ml) and incubated for 24 hrs, then pulsed with corresponding peptides at 100 µM and incubated for a further hour before being added to the remaining PBMCs. 3×10^5^ cells per well were cultured in R10 supplemented with recombinant human IL-7 (25 ng/ml) in 96 well U-bottom plates. The CTL lines were maintained by adding fresh R10 media every 3–4 days. Cells were fed on day 7 with recombinant IL-2 at a concentration of 25 U/ml. Class I HLA-matched or unmatched B cell lines pulsed with peptides were used as target cells, aliquoted at 5×10^4^ cells per well in 96-well U bottom plates. Effector cells were added to target cells at an effector/target (E∶T) ratio of 20∶1 unless otherwise specified. The amount of ^51^Cr released into the culture supernatants was quantified after 6 hrs of incubation, and the percent specific lysis was determined by using the following formula: [(*E-S*)/(*M-S*)]×100, where *E* is the experimental ^51^Cr release, *S* for spontaneous ^51^Cr release in the presence of culture medium and M for maximum release of incorporated ^51^Cr from target cells treated with 4% Triton X-100. The result was regarded as positive when recognition of the HIV target was >10% above the control.

## Results

### Subjects' background

Of 66 individuals recruited, 55 were female and 11 were male. At the time of enrolment, the median age was 29 years old (range 15–50), the CD4+ T cell count was 473 cells/µl (range 149–1,191) and the viral load was 4.24 log copies/ml (range 2.60–5.97). Table S1 shows HLA distribution.

### Frequency of Gag peptide responses and its HLA association

In this study we examined the CTL responses to 14 published Gag sequences to determine the extent of cross-clade reactivity in 66 Thai HIV-1 CRF01_AE infected individuals. Among 66 individuals, 29 individuals (43.9%) showed an IFN-γ response to at least one peptide in the ELISpot assay. In total, 79 responses were identified across all 14 peptides (Table S2). The most frequently recognized peptides were TV9 and FL12 recognised by 10 individuals, followed by p24_122–130_ PPIPVGDIY (PY9) and KY12 recognised by 9 and 8 patients, respectively. In contrast, p17_124_-p24_3_: SSSKVSQNYPIV (SV12) had the least number of responders, recognized by one individual, followed by NA8 and KR13 recognised by two and three patients, respectively.

Amongst the 79 responses detected in the ELISpot assay, 42 (53.2%) were responses against the 7 peptides that contained previously reported CRF01_AE epitopes. 52 of the 79 ELISpot responses (65.8%) were induced in patients carrying at least one HLA allele previously reported to restrict the tested epitopes. Of these, 28 out of 52 responses (53.8%) were induced in patients with HLA alleles known to restrict the tested CRF01_AE epitopes, and the remaining 24 responses (46.2%) were in patients carrying HLA alleles reported to restrict the epitopes in other subtypes. Furthermore, 18 out of these 24 responses (75%) were against peptides containing epitopes previously unreported in CRF01_AE infections, indicating that these are cross-clade reactive epitopes, while 6 out of these 24 responses (25%) were against peptides containing known CRF01_AE epitopes.

The remaining 27 out of 79 (34.2%) responses were in individuals carrying HLA alleles previously not known to restrict the tested epitopes, suggesting that at least one third of the peptide responses were restricted by unknown HLA alleles (Table S2). 19 out of these 27 responses were against the peptides containing epitopes previously unknown to be CRF01_AE epitopes, and the remaining 8 were against peptides containing reported CRF01_AE epitopes.

### Prediction of epitopes and their HLA restriction using an in silico model

**Figure 2:**
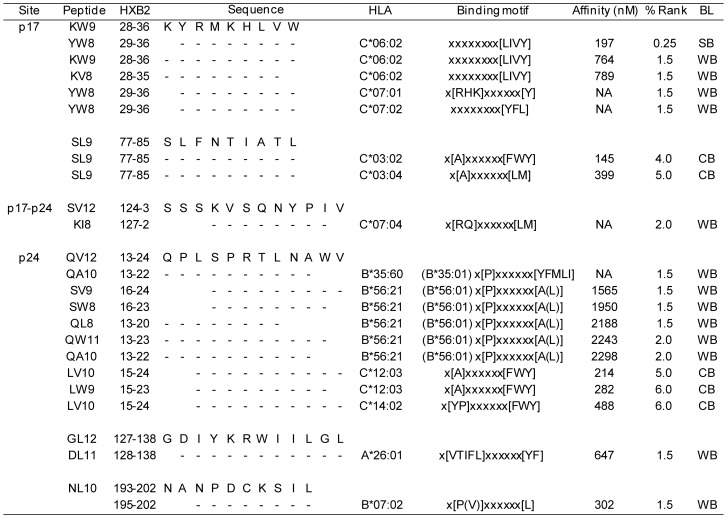
*In silico* epitope prediction for ELISpot responders carrying HLA alleles currently unknown to restrict CRF01_AE epitopes. We found 27 CRF01_AE specific CTL responses induced in patients carrying HLA alleles previously unknown to restrict CRF01_AE epitopes. Prediction of the optimal epitope within the peptide and its restricting HLA allele was performed using the *in silico* epitope prediction model HLArestrictor. In total, 19 epitope-HLA combinations were detected with binder levels defined as SB (Strong Binder), WB (Weak Binder), or CB (Combined Binder). NA: Not available, and BL: Binder level.

For the 27 responses induced in patients carrying HLA alleles previously not known to restrict the tested epitopes, we performed a prediction of the epitope and its restricting HLA allele using the latest peptide-binding motif based *in silico* program, HLArestrictor (Figure 2). In total, 19 dominant epitope candidates and their associated HLA alleles were detected amongst 6 peptides. Within these 6 peptides, the 2 peptides KW9 and SL9, which contained previously reported CRF01_AE epitopes, 7 dominant epitope candidates were detected, while we identified 12 epitope candidates and their associated HLA alleles in the remaining 4 peptides containing epitopes unreported in CRF01_AE (SV12, QV12, GL12, and NL10). 11 out of 19 (57.9%) epitope candidates were associated with HLA-C alleles, while 7 were associated with HLA-B alleles, and 1 was associated with an HLA-A allele. According to the binding affinity thresholds set by HLArestrictor, out of the 19 epitope-HLA complex candidates identified in our study, 1 was detected as SB, 13 as WB, and 5 as CB. Surprisingly, more than half (11/19) were associated with HLA-C alleles including SB, suggesting the possibility that there may be many HLA-C-associated epitopes that remain unreported.

### Identification of a novel epitope-HLA association in CRF01_AE infection with a ^51^Cr release assay

**Figure 3:**
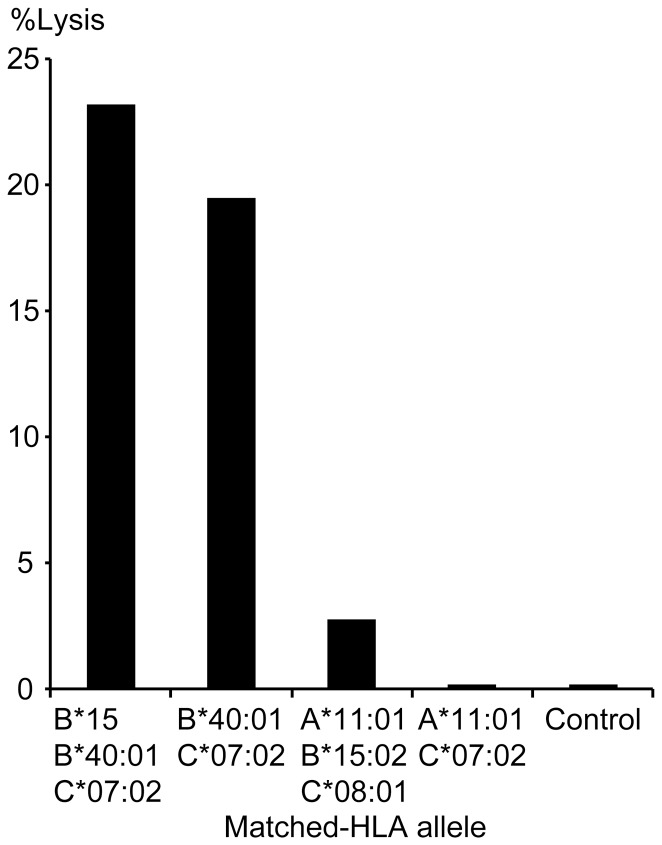
Cytotoxicity assay with T cells Demonstration of a novel epitope-HLA association by ^51^Cr release assay. Specific lysis of Gag peptide p24_122–130_ PPIPVGDIY (PY9) pulsed allogeneic target cells by effector CTLs from a HLA-B*40:01+ donor was assessed in a chromium release assay. The *Y* axis shows percentage specific lysis at an E∶T ratio of 20∶1 with the lysis (%) of unpulsed target cells subtracted. Effector cells were derived from patient 1509. HLA-B*40:01 matched cells pulsed with PY9 were also recognized by patient 326 (data not shown). HLA alleles shared by target cells and effector cells are shown; control indicates HLA-unmatched cells.

Using a ^51^Cr release assay, we further succeeded in demonstrating a novel HLA association of the CRF01_AE Gag epitope p24_122–130_ PPIPVGDIY (PY9), which had been previously reported to be restricted by HLA-B35 and B53 in clade A, B, C, D, CRF02_AG and HIV-2, but not in CRF01_AE. Significant lysis against the PY9 CRF01_AE variant was only detected in the presence of HLA-B*40:01-matched target cells, confirming HLA-B*40:01 as the restricting HLA allele (Figure 3). Out of 9 patients who made an ELISpot response to the peptide PY9, one patient carried the previously reported B7 supertype HLA-B*55:02, and two responders carried the newly detected HLA-B*40:01 allele (Table S2).

## Discussion

HIV-1 vaccines in clinical trials today are based on sequences derived from clades B, C, or A, but the identification of conserved HIV-1 CTL epitopes and an understanding of cross-clade CTL responses will be essential to broaden the vaccine responses to include other subtypes of HIV. In the present study, we performed the IFN-γ secreting CTL responses in a Thai cohort with CRF01_AE infection using a set of 14 well-established epitopes and designing CRF01_AE peptide analogues to these epitopes. Our Thai patients infected with CRF01_AE responded to all CRF01_AE analogues, which were not previously reported as CRF01_AE epitopes. Interestingly, however, these analogues were recognized by patients carrying HLA alleles that differed from those reported to restrict the published epitopes, exemplified by our confirmation of a novel HLA restriction of the p24 epitope PY9 by HLA-B*40:01 with a ^51^Cr release assay. Our findings indicate that the CTL recognition sites in HIV-1 Gag may be shared among different clades but these can be restricted by different HLA molecules, depending on the HLA polymorphism within the cohort.

The process of CTL activation is a highly sensitive and specific process, and a single mutation in the epitope can result in the lack of recognition by the CTLs, the impairment of peptide processing [19], [20], or the inhibition of the formation of peptide-HLA complexes [21], [22]. Therefore, the inter-clade and intra-clade sequence diversity of HIV-1 has been considered to be the primary barrier to the development of a globally effective vaccine. However, in this study, we have identified the cross-clade epitope candidates which had previously not been reported in CRF01_AE. 37 responses were found across 7 non-CRF01_AE epitopes, suggesting that these are novel cross-clade epitope candidates. It is noteworthy that although CRF01_AE is a recombinant HIV-1 with its Gag sequence derived from clade A (13.4% of Gag sequence discrepancy between clade A and CRF01_AE) [2], the three peptides SV12, NA8 and NL10 have not been reported in clade A.

Over the years, there has been much effort to identify HIV-1 epitopes that mediate potent cross-clade T cell responses [23], [24]. However, previous methods utilised peptide pools [23]–[26] or CTL clones with predetermined HLA alleles to observe cross-clade reactivity [27], [28]. The results of our study has shown at the level of single peptide, the lack of peptide recognition seen in the previous studies may have been due to the difference in HLA restriction allele among different population, rather than lack of CTL recognition.

Compared to clade B or C, there is far less epitope information available for clade A. This is the dominant clade circulating in eastern Europe, central Asia and eastern to central Africa, and given that HLA frequencies differ greatly between each region, we anticipate that a detailed epitope mapping study would further reveal the effect of HLA polymorphisms on a particular epitope's immunodominance and its association with viral control, as has been observed among other clades and ethnic groups [29], [30].

There are many reports of cross-clade reactivity in HIV-specific CTL responses [31]–[33]. The mechanism of epitope cross-clade reactivity is poorly understood, however it has been proposed that the more conserved the epitope, the more likely it is to instigate cross-clade reactivity [7], and the sequence variability at anchor positions of the HLA binding motif is thought to be the determining factor [2], [32]–[35]. However, our previous studies of ELISpot assays using overlapping peptides have shown that some peptides containing previously reported epitopes did not induce T cell responses in patients carrying HLA alleles known to restrict these epitopes even if their epitope sequences at anchor positions were compatible with the binding motif of the restricting HLA alleles [36]. The lack of a T cell response despite the binding motif matching with the epitope sequences may be accounted for by the amino acid sequence of the flanking regions, especially when the epitopes are shorter than the peptide tested, [19], [20] or sequence variation in positions other than the anchor regions, both of which may influence the recognition of the MHC-peptide complex by T cell receptors (TCRs) [21], [22].

Recently, *in silico* algorithms have been used to biometrically recombine and design vaccine epitopes that elicit CTL responses of higher breadth (number of peptide recognition) and depth (response of variants within an epitope) for experimental studies in rhesus macaques [37], [38]. Taken together with our data suggesting the potential of multiple epitopes with cross-clade reactivity, these studies support the possibility of the development of a cross-clade reactive vaccine.

In our study we were also able to use epitope prediction models to identify epitopes within peptides that induced responses in patients carrying HLA alleles previously unknown to restrict the tested CRF01_AE epitopes, highlighting the potential of such *in silico* models to identify epitopes restricted by rare HLA alleles like HLA-C. CTL-epitope information for HLA-C alleles has been sparse compared to A and B alleles, as can be observed in the Los Alamos database. The lower level of cell surface expression of HLA-C alleles compared to the other alleles [39]–[41] and the lack of protective HLA-C allele information in population studies [10]–[12] has hampered epitope mapping against HLA-C alleles. However, a recent single nucleotide polymorphism (SNP) study in a Caucasian cohort identified a region 35 kb upstream of the gene encoding the HLA-C molecule (−35(C/T)) as the second strongest determining factor for viral control [42], capable of inducing either a higher (C) or lower (T) expression level of HLA-C on the cell surface [43]. As seen from the recent identification of dominant HLA-C-restricted epitopes [44], we can expect an increasing number of studies extending our understanding of how the HLA-C alleles contribute to viral control. Epitope information from HLA-C alleles will also contribute to our knowledge of a given epitope's cross-clade reactivity or clade-specificity. It is warranted to study further especially to demonstrate the potentially novel HLA alleles restriction listed in Figure 2.

As a post hoc analysis, this study also has several limitations. Firstly, due to limited PBMCs available from the patients we focused on Gag peptides and did not investigate responses to whole viral proteins. However, Gag, especially the p24 protein, is one of the most important target antigens for viral control [36], [45], [46], due to their role in the selection of escape mutations that lead to viral fitness costs [47], its sequence stability compared to other viral particles [1], [2], [7], the abundance of the protein on incoming virions [48], as well as its rapid antigen presentation following viral infection [49]. For the development of a globally effective CTL-induced vaccine, detailed mapping of Gag epitopes and their restricting HLA alleles will be essential. Secondly, in this study we analysed 66 subjects, but further studies with larger population sample sizes may help identify cross-clade CTL epitopes restricted by minor HLA alleles and allow us to differentiate linkage disequilibrium effects from true associations. Thirdly, since the CTL epitope information was limited when these 14 peptides were designed in the year 2000, this peptide selection may not be optimal in the current setting. However, even after 2000, surprisingly only three CRF01_AE-associated epitopes with responsible four digits HLA allele were reported according to the latest Los Alamos database 2012; p24_35–43_ EVIPMFSAL restricted by HLA-A*26:01 and A*26:03 [50], p24_145–153_ YSPVSILDI by HLA-C*01:02 [51], and p24_209–217_ ATLEEMMTA by HLA-A*02:06 [52]. This is why we think that our data is still worth reporting. Fourthly, we used five 12-mer peptides and one 13-mer peptide in this study to maximize the frequency of responses by spanning more epitopes. However, this extension of the peptides may have lowered the peptide responses but we did not find an obvious tendency that the longer peptides had less response. For optimal epitope and responsible HLA confirmation, further experiment with peptide narrow down will be required.

Fifthly, in computational epitope prediction, although more than half of candidates were detected from HLA-C alleles, prediction of HLA-C allele-associated epitopes was not accurate compared to that of HLA-A or B-associated as commented by programmers [18]. It is warranted to demonstrate the potentially novel HLA alleles restriction listed in Figure 2.

From our findings, we conclude that the HLA restriction of an epitope in a given population is dictated by two factors: HLA polymorphism within the population and viral sequence diversity. Previous studies on CTL cross-recognition have focused on the sequence divergence between the clades, and have promoted the inclusion of highly conserved epitopes in CTL-epitope vaccines [7], [32]–[35]. However, we have shown that in different cohort populations, CTL recognition of the same epitopes may occur through unique HLA restrictions. We believe that the HLA restriction of epitopes should be determined for a given population prior to the selection of vaccine candidate immunogens, as certain epitopes will be able to induce broad, cross-clade responses which will increase the potential efficacy of the vaccine in the given population. We hope that the novel epitopes and HLA restrictions identified in this study will contribute to the development of a cross-clade reactive HIV vaccine.

## Supporting Information

Table S1 **HLA distribution among 66 HIV-1 CRF01_AE-infected Thais.** HLA distribution by population frequency is shown.
(XLSX)

Table S2 **HLA allele information of ELISpot assay responders and its compatibility with previous report.** In total, 79 responses among 14 epitopes were identified. HLA allele information of ELISpot assay responders and its compatibility with previous report of responsible HLA alleles listed in Los Alamos database are shown.
(XLSX)
