# Correction: The Effect of HLA Polymorphisms on the Recognition of Gag Epitopes in HIV-1 CRF01_AE Infection

**DOI:** 10.1371/journal.pone.0106978

**Published:** 2014-08-22

**Authors:** The PLOS ONE Staff

There is an error in the data in cell Y47 of the Supplementary Table 2. It should read C*03:04. Please see the corrected Table S2 below.

## Supporting Information

Table S2 **HLA allele information of ELISpot assay responders and its compatibility with previous report.** In total, 79 responses among 14 epitopes were identified. HLA allele information of ELISpot assay responders and its compatibility with previous report of responsible HLA alleles listed in Los Alamos database are shown.
(XLSX)
